# Human Milk–A Valuable Tool in the Early Days of Life of Premature Infants

**DOI:** 10.3389/fped.2019.00266

**Published:** 2019-07-09

**Authors:** Ekhard E. Ziegler

**Affiliations:** ^1^The University of Iowa, Iowa City, IA, United States; ^2^Department of Pediatrics, University of Iowa, Iowa City, IA, United States

**Keywords:** human milk, early, premature infant, parenteral nutrition, avoidance of nutrient deficit

## Abstract

The objective of early premature infant nutrition is to maintain, during the turbulent early days of life, a flow of nutrients that differs only minimally from that which would have prevailed had the infant remained *in utero*. Out of necessity, nutrients have at first to be provided mainly via the parenteral route. While that is going on, the feeding of small amounts of human milk (gut priming) is initiated as soon as practical. As mother's own milk is not available in sufficient quantity at this time, donor milk needs to be used temporarily. If not available, formula should be used. Gastric residuals are physiologic at this stage and are monitored to guide the increase of the size of feedings. As the volume of milk is gradually increased, nutrient fortification is initiated when the milk volume reaches around 20 ml/kg/day. There is no need to start with less than full-strength fortification. Fortification should employ one of the liquid fortifiers. Adjustable fortification may be employed but is labor-intensive and is not a necessity as long as full feeding volumes of around 170 ml/kg/day are maintained. As the infant grows beyond 1,500 g the level of fortification can be reduced gradually by omitting fortification first from one, and then from more feedings. After discharge there is still a need for fortification, which requires the mother to express some of her milk so it can be fortified. Nutrient supplementation directly to the infant would obviate the need for milk expression.

## Introduction

There is no time in the premature infant's life that is more important than the first few days. It is a time when the provision of nutrients meets with technical difficulties and nutrient intakes frequently fall short of needs. It is a time when the rich flow of nutrients that has been supporting rapid growth and development *in utero* has just been cut off abruptly. The trauma of this disruption is intense and has severe consequences unless the flow of nutrients is restored promptly. Restoring the flow of nutrients is challenging, technically, and otherwise, and that is the reason why prompt restoration is so often not occurring. But this is a time of exquisite vulnerability to the lack of adequate nutrients and prompt restoration is mandatory. Failure to provide adequate amounts of protein and energy during this early period has serious consequences (1). Whether and to what extent nutrient deficits can be made up later is not known exactly, but the widespread occurrence of impaired cognitive development among former preterm infants suggests that the ability to make up is limited (2). Fortunately, we have the techniques for promptly restoring the flow of key nutrients. If nevertheless the restoration of nutrient intakes does not always occur in a timely fashion, it is because of misperceptions and misinterpretation of physiologic signs, such as gastric residuals. While restoration of the nutrient flow has absolute primacy, it is closely followed in urgency by the need to establish enteral feedings. This is where human milk with its unique properties plays a crucial role, a role that is not always fully utilized.

The benefits of human milk for the premature infant are well-established. They are of particular importance during the early days and weeks of life. The property of human milk that makes it so valuable at this time is its strong trophic effect on the immature gut. This maturational effect not only enables earlier establishment of full feedings, it also provides protection against necrotizing enterocolitis (NEC) and against late-onset sepsis. But for these effects to come to bear, human milk must be fed, and not be withheld, as is too often the case, for various reasons. That is why sound feeding practices are of such crucial importance during the early days of life. One essential condition is the acceptance of gastric residuals as what they are, namely mere manifestations of gut immaturity rather than as signs of “feeding intolerance” or, worse, “impending NEC.” Gastric residuals eventually subside, the faster so if the necessary stimulation by feeding of human milk is provided. The only disadvantage of human milk is its low nutrient content relative to the high needs of the premature infant that must be overcome by nutrient fortification.

The objective of the present essay is to foster feeding practices that maximize the utilization of the beneficial effects of human milk. Good feeding practices are part of the overall effort to minimize the hazards that premature birth presents to the baby. The present discussion makes the assumption that parenteral nutrition is being provided starting within hours of birth, that it is providing adequate amounts of amino acids and energy, and that it is not terminated until enteral nutrition has reached near-full levels.

The opinions offered are derived largely from personal experience and anecdotal reports, but are overall consistent with the current literature.

To begin with, it is useful to review some basic facts about the preterm infant and to agree on some of the principles that govern nutrition of the premature infant:

1. The amounts of nutrients the fetus is receiving are known and serve as a model of the nutrient needs of the preterm infant.
2. The flow of nutrients to the recently born preterm infant should not be interrupted for more than a few hours.
3. The gastrointestinal (GI) tract of the preterm infant is at birth in an immature state and incapable of handling a full load of nutrients.
4. If properly stimulated (primed), the GI tract acquires, in a matter of days, the functional maturity for accepting and absorbing full nutrients.
5. While the GI tract is immature, maintaining an adequate supply of nutrients requires the use of parenteral nutrition.
6. The GI tract is susceptible to necrotizing enterocolitis (NEC) and is presumed to remain so for a while even after acquiring adequate motility.
7. The risk of NEC is not modified by the volume and timing of feedings.
8. The risk of NEC increases if formula instead of human milk is fed.
9. The infant should incur no weight loss except that which is explained by the birth-related shrinking of the extracellular fluid space.
10. If weight loss is greater, every effort must be made to recover lost ground as quickly as possible.

## Parenteral Nutrition

As the gut is at the outset unable to absorb nutrients, the provision of nutrients must of necessity be via the parenteral route, which has been shown to be effective and safe (3). Parenteral nutrition administered through an umbilical vessel is initiated within a few (preferably <2) hours of birth. It should provide protein/amino acids of at least 2.0 g/kg/day but preferably 3.0 g/kg/day. Blood glucose concentration must be monitored at regular intervals, and hyperglycemia and hypoglycemia, if present, must be treated appropriately. Monitoring of blood urea is not necessary, but monitoring of acid-base status is. After a few days, the administration of parenteral nutrition is shifted to a peripherally placed central venous catheter. As the volume of enteral feedings increases, parenteral nutrition is tapered while maintaining at all times an adequate total nutrient intake. Parenteral nutrition should not be discontinued prematurely as that practice leads to a temporary slowdown or cessation of growth. Parenteral nutrition should be continued until enteral feeds have reached at least 90% of full feedings.

## The Initiation of Feedings (Gut Priming)

The main reason for the common hesitation to provide enteral feedings during the early days of life is the frequent occurrence of gastric residuals. It is therefore, important to recognize that residuals are merely a manifestation of immaturity of the GI tract and have nothing to do with NEC. Immature motility needs to be overcome by GI priming, which is done better by human milk than by anything else. Until immaturity is overcome, full feedings are not possible. The manifestations of gut immaturity are physiologic and subside in time with proper management. The risk of NEC, while ever present, is not eliminated by withholding of feedings.

The sole purpose of providing enteral feedings early on, also known as trophic feedings or gut priming, is to facilitate maturation of the immature gastrointestinal tract. The progress of gut maturation is, for lack of a better parameter, judged by the decrease of the size of gastric residuals. The goal of gut priming is to reach a rate of gastric emptying that permits the administration of full or near-full feedings. Until that goal is reached, parenteral nutrition must be continued to ensure an adequate supply of nutrients at all times. In practice, the initiation of feedings is often delayed by one or more days after birth due to the belief that the risk of NEC is thereby reduced. Practice for many years has shown that such a delay does not reduce the risk of NEC and only causes a delay in reaching full feeds. Thus, gut priming should always be initiated within a few hours of birth, at the latest within the first 24 h of life, and should not be interrupted due to the occurrence of gastric residuals.

There is no evidence that the frequency of early feedings or the strength of feedings are of importance. What is important is that gastric residuals are monitored because the rate of advancement of feeds needs to be based on the occurrence and size of gastric residuals. Historically, before the phenomenon of gut immaturity was fully appreciated, feedings were often advanced too rapidly, and the ensuing gastric residuals were interpreted as constituting NEC grade I (incipient NEC) and prompted the withholding of feedings for 24 h or longer. Today we know that gastric residuals are physiologic, as long as they are of modest size and of normal color, and may require a reduction in the size of feedings but not a cessation of feedings. There is no evidence that the volume of feedings or the rate of advancement of feedings impacts the risk of NEC.

The choice of feeding for GI priming is important because human milk offers several strong advantages, including that it enhances the maturation of the GI tract. Every effort must therefore be made to secure the mother's milk for gut priming. Freezing of milk entails loss of all cellular components and diminution of some of the valuable components of human milk. But the logistics of providing fresh milk are such that much milk is provided after a shorter or longer period of freezing.

Since the availability of mother's own milk is typically limited in the first few days after birth, the temporary (until mother's milk comes in) use of donor milk is necessary. Donor milk is pooled milk from multiple donors who are typically in advanced stages of lactation with its decreases of some components of breast milk. It is always frozen and heat treated, which causes a decrease of the content of nutrients and other components, and the complete elimination of components such as lipase. Despite these decreases, donor milk is the preferred feeding during the first few days after birth when mother's own milk is not available in sufficient quantity. It is also the preferred feeding whenever a mother experiences diminishing milk supply in spite of efforts to maintain her milk supply, which, unfortunately, is a rather common occurrence. If donor milk is not available, formula is a second choice for gut priming. NEC is extremely uncommon in the first few days of life and the feeding of formula temporarily is preferred over not giving any feedings at all.

## Fortification of Human Milk

Growth and nutrient needs of premature infants are shown in Table 1. Infants weighing <1,200 g require an intake of protein of around 4.0 g/kg/day in order to grow like the fetus. If human milk is fed at 150 ml/kg/day, it provides only about 2 g/kg/day of protein (except during the first 2 weeks of lactation when the protein content of human milk tends to be higher). The need for extra protein is thus evident. Although their requirements are not shown in Table 1, the intakes of electrolytes, minerals, and vitamins from human milk are similarly not adequate to cover the needs of the small premature infant. Most of the nutrients in short supply are provided by fortifiers. Powder fortifiers have been available for some time but were woefully inadequate mainly in the amount of protein they provide. If used, the addition of extra protein is mandatory (4). The newer liquid fortifiers provide more protein so that the total intake from fortified human milk approaches the required 4.0 g/kg/day.

**Table 1:** Estimated protein intakes needed for weight gain like the fetus.

**Body weight (g)**	**500–700**	**700−900**	**900–1,200**	**1,200–1,500**	**1,500–1,800**	**1,800–2,200**
Fetal weight gain (g/d)	13	16	20	24	26	29
(g/kg/d)	21	20	19	18	16	14
**Protein (g/kg/d)**						
Loss	1.0	1.0	1.0	1.0	1.0	1.0
Growth (accretion)	2.5	2.5	2.5	2.4	2.2	2.0
Required intake
Parenteral	3.5	3.5	3.5	3.4	3.2	3.0
Enteral	4.0	4.0	4.0	3.9	3.6	3.4
**Protein/Energy (g/100 kcal)**						
Parenteral	3.9	3.8	3.5	3.1	2.9	2.7
Enteral	3.8	3.7	3.4	3.1	2.8	2.6

### Initiation of Fortification

Fortification with a liquid multicomponent fortifier should be initiated when the feeding volume reaches around 25 ml/day, which has been the practice at the author's institution for many years. There is nothing gained by waiting any longer, nor is there any advantage from starting at less than full strength fortification. Concerns about an increase in osmolality of feedings caused by the fortifier are unfounded. It has been the practice in many NICU's to initiate fortification when feedings reach a volume of around 100 ml/kg/day. The origin of this practice is murky as is its rationale. The disadvantage from delaying fortification is a deficit in nutrient intakes and a delay in the achievement of a full nutrient intake. Whether the early addition of fortifier delays the attainment of full feedings is a matter of debate. Individualized fortification, if used, is usually initiated only after the first week of life. Fortification with one of the liquid fortifiers in standard fashion is recommended as individualized methods have not been shown to yield superior nutrient intakes.

## Monitoring of Growth

As growth provides the ultimate proof of nutritional adequacy (or inadequacy), the monitoring of growth assumes central importance. It is complicated by the fact that all infants undergo a physiologic weight loss, which is not caused by nutritional inadequacy but which is due to constriction of the infant's body water after birth. The decrease of body water is somewhat variable but usually is equivalent to about −0.6 weight z-scores. A loss of 0.6 weight z-scores is therefore physiologic, but any greater weight loss is abnormal and is associated with a risk of neurocognitive impairment.

**Figure 1:**
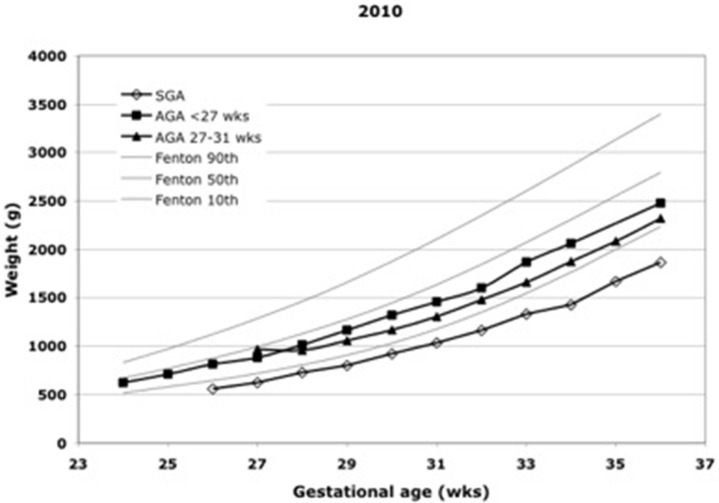
Growth of premature infants.

**Figure 2:**
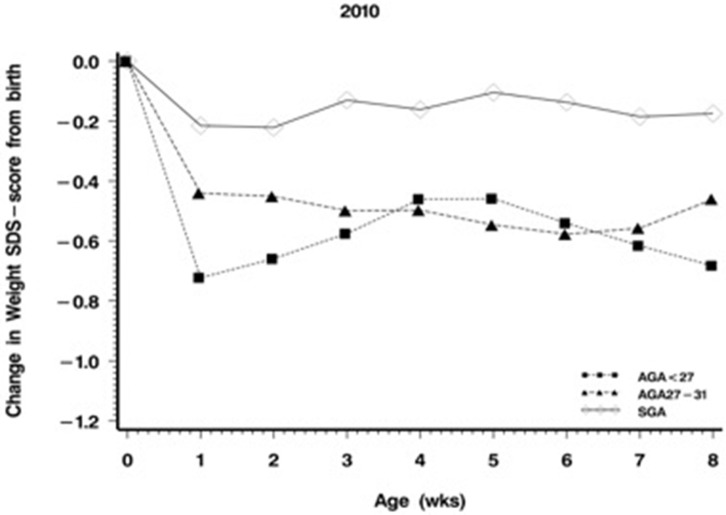
Growth of premature infants.

Daily weight is routinely monitored in every unit, and length and head circumference are measured periodically in most units. The problem lies in the interpretation of measurements. The calculation of average weight gain over some period of time is not an appropriate method because normative values decrease with increasing body weight and are generally not known. An acceptable way of interpreting growth measurements is to display them on charts of fetal growth, where growth in parallel to percentile lines is the expected norm (Figure 1). A fall-off from percentile lines indicates growth failure. The preferred way to interpret growth measurements is to transform them into z-scores derived from current references of fetal growth (5) and to express them as deviations from the birth z-score (Figure 2). The weight z-score should not drop by more than 0.6, and if it does, every effort should be made to make up the deficit as soon as possible. The z-scores for length and head circumference should not drop at all.

The widespread notion that some degree of postnatal weight loss is unavoidable is slowly giving way to the notion that loss of more than water is avoidable and loss of water only should be the norm. Examples of avoidance of weight loss are provided by Senterre and Rigo (6).

## Tapering of Fortification

Human milk fortified with a liquid fortifier meets approximately the high nutrient needs of the infant weighing <1,500 g. As the infant grows beyond 1,500 g, this regimen provides a surfeit of nutrients that becomes larger as the infant grows larger. While no adverse consequences of the nutrient surfeit in the short run are known, it is counterintuitive to burden the infant with an excess of nutrients. An excess of a comparable size is not routinely provided to any other group of infants. And, if data in full term infants may be used as guidance, the provision of unduly high intakes of protein in early life has been shown to lead to an increase in adiposity and to a greater risk of obesity later in life (7). It is, of course, not known whether the same mechanism may be at work in premature infants, but prudence would suggest that an excess of protein ought to be avoided.

It is suggested that nutrient intakes be reduced as the infant grows. There are no established methods or schedules for the tapering of fortification. A decrease of nutrient intakes could be brought about by diminishing the amount of fortifier added to each feeding. Alternatively, omitting fortification from some feedings and not from others seems to be a simpler way of accomplishing tapering. A suggested schedule would be to omit fortification initially from one feeding per day, then from two feedings per day, and so forth, until at the time of discharge only about 30% of feedings are fortified.

### Transition to Breastfeeding

Infants to be discharged from the hospital must be able to feed by mouth. The transition to breastfeeding can be a protracted process. It is sometimes aided by the use breast shields.

## Fortification After Discharge

The usual criteria of discharge readiness include the ability to take all feedings by mouth, whether it is directly from the breast or from a bottle. This readiness is usually reached, in the absence of complications, before the infant's weight reaches 3 kg. Because mother's milk alone does not meet the nutrient needs of infants weighing 3 kg or less, fortification is needed after discharge. Furthermore, infants at discharge are often undergrown and thus have additional nutrient needs for catch-up growth. Post-discharge fortification schemes vary and range from full fortification to token fortification with small amounts of protein. A rational approach would be to fortify two feedings per day in the case of a liquid fortifier, and three feedings per day if a powder fortifier is used. How long fortification should continue is not settled, but a reasonable point at which to discontinue fortification would be the attainment of a weight of 4 kg.

Post-discharge fortification places a large burden on the infant's mother as she needs to express her milk for a portion of the infant's daily consumption. It would be a major advantage if the supplemental nutrients were to be available in the form of a liquid that could be administered in small volume directly to the infant. The infant could then receive all its milk directly from the breast. Unfortunately, such a nutrient solution is not commercially available and would be difficult to prepare in the home.

## Conclusion

For human milk to exert all its benefits for the premature infant, it has to be fed right from birth when its trophic effects are of crucial importance. As gastric residuals decline and human milk feedings progress, nutrient fortification must be initiated. Weight monitoring must ensure that postnatal weight loss never exceeds −0.6 weight z-scores. As the infant's nutrient needs decline, the level of human milk fortification can be tapered. But at discharge most infants still require nutrient fortification.

## Author Contributions

The author confirms being the sole contributor of this work and has approved it for publication.

### Conflict of Interest Statement

The author declares that the research was conducted in the absence of any commercial or financial relationships that could be construed as a potential conflict of interest.
